# Accuracy of eight deformable image registration (DIR) methods for tomotherapy megavoltage computed tomography (MVCT) images

**DOI:** 10.1002/jmrs.236

**Published:** 2017-07-29

**Authors:** Wannapha Nobnop, Hudsaleark Neamin, Imjai Chitapanarux, Somsak Wanwilairat, Vicharn Lorvidhaya, Taweap Sanghangthum

**Affiliations:** ^1^ Department of Radiologic Technology Faculty of Associated Medical Sciences Chiang Mai University Chiang Mai Thailand; ^2^ Division of Radiation Oncology Department of Radiology Faculty of Medicine Chiang Mai University Chiang Mai Thailand; ^3^ Division of Radiation Oncology Department of Radiology Faculty of Medicine Chulalongkorn University Bangkok Thailand

**Keywords:** adaptive radiotherapy, deformable registration, head and neck, radiotherapy

## Abstract

**Introduction:**

The application of deformable image registration (DIR) to megavoltage computed tomography (MVCT) images benefits adaptive radiotherapy. This study aims to quantify the accuracy of DIR for MVCT images when using different deformation methods assessed in a cubic phantom and nasopharyngeal carcinoma (NPC) patients.

**Methods:**

In the control studies, the DIR accuracy in air‐tissue and tissue‐tissue interface areas was observed using twelve shapes of acrylic and tissue‐equivalent material inserted in the phantom. In the clinical studies, the 1st and 20th fraction MVCT images of seven NPC patients were used to evaluate application of DIR. The eight DIR methods used in the DIRART software varied in (i) transformation framework (asymmetric or symmetric), (ii) DIR registration algorithm (Demons or Optical Flow) and (iii) mapping direction (forward or backward). The accuracy of the methods was compared using an intensity‐based criterion (correlation coefficient, CC) and volume‐based criterion (Dice's similarity coefficient, DSC).

**Results:**

The asymmetric transformation with Optical Flow showed the best performance for air‐tissue interface areas, with a mean CC and DSC of 0.97 ± 0.03 and 0.79 ± 0.11 respectively. The symmetric transformation with Optical Flow showed good agreement for tissue‐tissue interface areas with a CC of (0.99 ± 0.01) and DSC of (0.89 ± 0.03). The sequences of target domains were significantly different in tissue‐tissue interface areas.

**Conclusions:**

The deformation method and interface area affected the accuracy of DIR. The validation techniques showed satisfactory volume matching of greater than 0.7 with DSC analysis. The methods can yield acceptable results for clinical applications.

## Introduction

Megavoltage computed tomography (MVCT) images are acquired daily in the helical tomotherapy unit (Tomotherapy Inc., Madison, Wisconsin, USA) with the primary purpose of more accurate target localisation^1^ and can also be used for daily dose computation.^2^ The ability to monitor inter‐fractional anatomical variations is a prerequisite to enable plan adjustments which account for discrepancies, changes in target volume and organs at risk. As head and neck cancer patients may undergo significant anatomical changes over a 6‐ to 7‐week course of radiation treatment for various reasons, any volume shrinkage near the facial surface is likely to cause migration of the radiation‐sensitive parotid glands towards high‐dose regions.^1^ This might result in unforeseen changes in delivered dose such as non‐uniform coverage of the target volumes and increased dose to the organs at risk (OAR).^3^ Ideally, when a patient's anatomy changes, a new adaptive plan must be developed. The procedure includes the modification of an initial plan according to the changes in target volume or normal organs, commonly known as adaptive radiation therapy (ART).^4^ The most important part of routine use of ART is accurate deformable image registration (DIR) using verification imaging to create the automatic contour and calculated accumulated doses.^5^

Regarding DIR accuracy, it is generally understood that the use of kilovoltage computed tomography (kVCT) will allow more accurate deformation by the DIR algorithm. The DIR will work best with feature‐rich images where there is little or no ambiguity between corresponding points in the source and target images.^6^ MVCT image registration accuracy is limited by low image contrast.^7^ Moreover, the results of the registration naturally depend on the deformation model. The choice of deformation algorithms and transformation frameworks in MVCT images is of great importance in the registration process, as that entails an important compromise between computational efficiency and richness of description.^8^ The popular tool‐kit for image registration, Deformable Image Registration and Adaptive Radiotherapy (DIRART) is a software suite for DIR plus ART. DIRART is a large set of programs developed using MATLAB. DIRART works in a complementary fashion with Computational Environment for Radiotherapy Research (CERR) to offer additional function.^9^ DIRART provide the capability of selecting various deformation algorithms, transformation frameworks and mapping directions for deformable registration procedures.

Deformable image registration attempts to provide the mapping between volume elements in one image to the corresponding volume between two different image sets: the source and target images. There are many automated DIR algorithms that can provide a mapping or deformation vector field (DVF) between two images.^10^ Regarding the transformation frameworks, *the asymmetric transformation* constitutes the majority of the existing registration algorithms. As a consequence, when interchanging the order of input images, the registration algorithm does not estimate the inverse transformation. The statistical analysis that follows registration is biased on the choice of the target domain, whereas *symmetric transformation* simultaneously estimates both the forward and backward transformations. The data matching term quantifies how well the images are aligned when one image is deformed by the forward transformation and the other image is deformed by the backward transformation.^8^ For the deformation algorithms, *Original Demons* and *Original Horn & Schunck Optical Flow* are non‐parametric deformation algorithms based on a vector per voxel method that describes the displacement to model the deformation of the anatomy for well‐studied models of fluid flow or the deformation of a viscoelastic material.^11^ Yeo et al.^6^ demonstrated that the Optical Flow algorithm can perform accurate DIR in low contrast regions. Demons is a well‐known algorithm for intensity‐based DIR.^12^ Both the Optical Flow and Demons algorithms have been used in deformable registration for commercial software.^6^, ^12^ Regarding the mapping direction, when the *forward mapping* is estimated, every voxel of the source image is pushed forward to its estimated position in the target image. When the *backward mapping* is estimated, the pixel value of a voxel in the target image is pulled from the source image.^8^ Therefore, the sequences of the target domain were the effect of the deformable registration process.

Many deformation models are used for image registration because the suitability of application of a particular evaluation metric in validating DIR is dependent on the clinical deformation observed.^13^ We are interested in answering the question “How well do the different DIR methods perform in tomotherapy MVCT images for nasopharyngeal carcinoma (NPC) patients?” This study aims to evaluate the accuracy of DIR on MVCT images of phantom and NPC patients using DIRART software and various (i) transformation frameworks, (ii) DIR registration algorithms and (iii) mapping directions.

## Methods

### Phantom and patients

**Figure 1:**
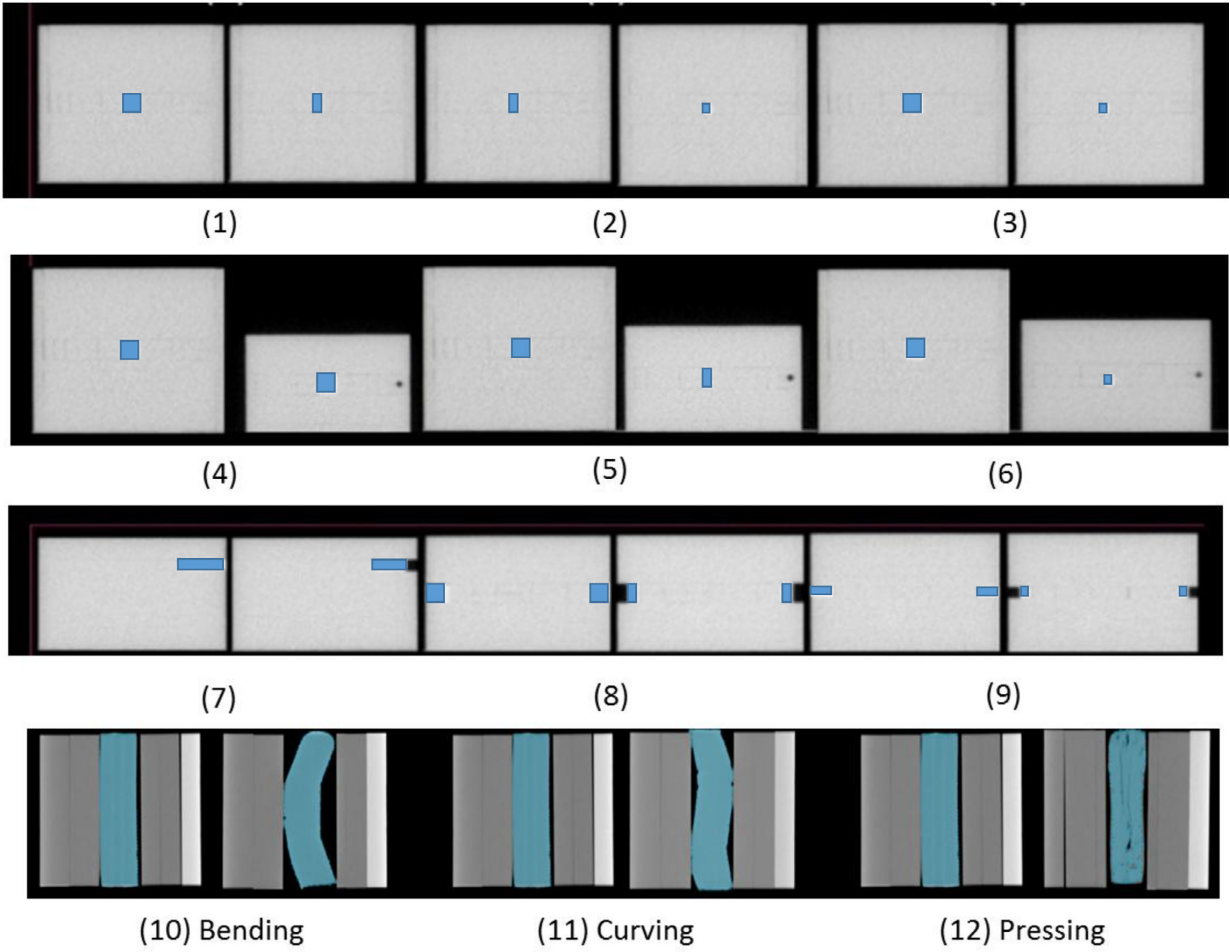
The source and target images of the deformation of twelve shapes. An acrylic material was inserted in a cubic phantom to simulate the volume changes in tissue‐tissue interface areas (cubic no. 1–6) and air‐tissue interface areas (cubic no. 7–9). Tissue equivalent materials in bent, curved and pressed shapes (cubic no. 10–12) were inserted in the cubic phantom to simulate the non‐rigid volume changes in air‐tissue interface areas.

To simulate the deformation in head and neck cancer, the deformable investigation areas in a phantom and clinical cases were divided into two groups: tissue‐tissue and air‐tissue interfaces. Twelve shapes in the source and target images are shown in Figure 1. The acrylic materials (density 1.15 g/cm^3^) and superflab tissue equivalent materials (diethylhexyl phthalate: DEHP, density 1.02 g/cm^3^) were inserted in a cubic phantom. The images simulated the target/OAR volume changes in tissue‐tissue interface areas (cubic no. 1–6) and air‐tissue interface areas (cubic no. 7–9). The tissue equivalent materials in bent, curved and pressed shapes (cubic no. 10–12) were inserted in the cubic phantom to simulate the non‐rigid volume changes in air‐tissue interface areas.

In the clinical cases, this study had institutional ethics approval with the study code: RAD‐2559‐03998/Research ID: 3998 from the Research Ethics Committee. Helical tomotherapy (HT) treatment planning from seven nasopharyngeal carcinoma patients was randomly selected in this study. All treatment plans prescribed a dose of 70 Gy delivered in 33 daily fractions.

### Image data acquisition

MVCT images of the source and target of twelve shapes were assessed in normal scan mode using the helical tomotherapy unit with a voxel size of 0.763 × 0.763 mm^2^ and slice thickness of 4 mm.

The 4‐mm slice thickness MVCT images of seven NPC patients at the first treatment fraction were acquired using the helical tomotherapy unit as the source image, and the 20th fraction was acquired as the target image.

### Target delineation

The twelve shapes of acrylic and superflab materials inside the cubic phantom were localized before deformation (source images) and after deformation (target images as the reference). The automatically deformed contour was generated by DIRART software and compared with the reference contour.

In NPC cases, the target and organ at risk (OAR) were defined by the radiation oncologist for treatment planning. The contours of both parotid glands were transferred to the first day MVCT images as the source images for the deformable investigation of the air‐tissue interface.

Both parotid glands on the 20th MVCT images were contoured by the same oncologist who localized the target and OAR. These contours were compared to the automatically deformed structure generated by the deformable image registration software.

### Deformable image registration

A deformable image registration using DIRART version 1a developed by Yang (2009)^9^ was used to create automatically deformed contours of the deformation of twelve acrylic shapes and tissue equivalent materials and the 20th MVCT images of seven NPC patients. The registration used the different deformation models. The eight DIRs methods were performed using DIRART software with various (i) transformation frameworks (asymmetric or symmetric transformation), (ii) DIR registration algorithms (Demons or Optical Flow algorithms) and (iii) mapping directions (forward or backward mapping). Details of the methods are shown in Table 1.

**Table 1:** Summary of eight deformable image registration methods with varying (i) transformation frameworks, (ii) DIR registration algorithms and (iii) mapping directions

No.	DIR methods^1^	Transformations		DIR algorithms		Mapping directions	
		Asymmetric	Symmetric	Optical flow	Demons	Backward	Forward
1	Asy‐OF_BW_	X		X		X	
2	Asy‐OF_FW_	X		X			X
3	Asy‐DM_BW_	X			X	X	
4	Asy‐DM_FW_	X			X		X
5	Sym‐OF_BW_		X	X		X	
6	Sym‐OF_FW_		X	X			X
7	Sym‐DM_BW_		X		X	X	
8	Sym‐DM_FW_		X		X		X

^1^Asy‐OF_BW_, asymmetric transformation with the Horn and Schunck optical flow algorithm and backward mapping; Asy‐OF_FW_, asymmetric transformation with the Horn and Schunck optical flow algorithm and forward mapping; Asy‐DM_BW_, asymmetric transformation with the Demon algorithm and backward mapping; Asy‐DM_FW_, asymmetric transformation with the Demon algorithm and forward mapping; Sym‐OF_BW_, symmetric transformation with the Horn and Schunck optical flow algorithm and backward mapping; Sym‐OF_FW_, symmetric transformation with the Horn and Schunck optical flow algorithm and forward mapping; Sym‐DM_BW_, symmetric transformation with the Demon algorithm and backward mapping; Sym‐DM_FW_, symmetric transformation with the Demon algorithm and forward mapping.

To establish the optimum DIR performance for each algorithm, various parameters were systematically adjusted: four multigrids were used (*n* = 1, 2, 3 and 4) with 10*n*–40*n* iterations per pass,^6^ the number of passes for the Optical Flow algorithm was 6, and that of the Demons algorithm was 2–6. Coarser stages were typically run with a greater number of passes to improve the agreement with the target image prior to resampling at finer resolutions.^6^

### Validation techniques

The accuracy of the methods was compared using an intensity‐based criterion (correlation coefficient, CC)^11^ and volume‐based criterion (Dice's similarity coefficient, DSC).^5^

The correlation coefficient can be between −1 and +1. The value +1 represents a maximum correlation between the images.^11^ For the volume‐based criterion, the most common overlap metric is the DSC metric that computes the number of pixels that overlap between the two volumes. If the images have no overlap, the DSC is 0, and if the contours become identical, the DSC approaches a value of 1.^5^ Goldberg–Zimring et al.^14^ suggested that satisfactory volume matching should be 70% or greater for adaptive radiotherapy applications. An analysis of variance (ANOVA) using SPSS statistical software version 17 was used to assess the impact of each DIR method.

## Results

### The deformation in tissue‐tissue interface areas

**Figure 2:**
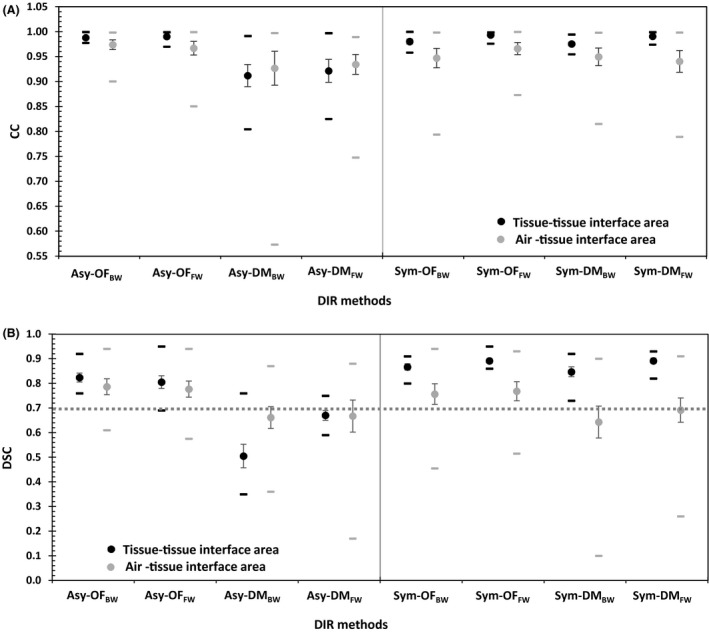
The mean (circles), standard error (vertical lines) and range (horizontal lines) of the (A) correlation coefficient (CC) and (B) Dice's similarity coefficient (DSC) for eight DIR methods^†^ in tissue‐tissue interface and air‐tissue interface areas. ^†^Asy‐OF_BW_, asymmetric transformation with the Horn and Schunck optical flow algorithm and backward mapping; Asy‐OF_FW_, asymmetric transformation with the Horn and Schunck optical flow algorithm and forward mapping; Asy‐DM_BW_, asymmetric transformation with the Demon algorithm and backward mapping; Asy‐DM_FW_, asymmetric transformation with the Demon algorithm and forward mapping; Sym‐OF_BW_, symmetric transformation with the Horn and Schunck optical flow algorithm and backward mapping; Sym‐OF_FW_, symmetric transformation with the Horn and Schunck optical flow algorithm and forward mapping; Sym‐DM_BW_, symmetric transformation with the Demon algorithm and backward mapping; Sym‐DM_FW_, symmetric transformation with the Demon algorithm and forward mapping.

The DIR accuracy results were consistent between the similarity metric (CC) and overlapping analysis (DSC). Figure 2 shows the (A) CC and (B) DSC of eight DIR methods in tissue‐tissue interface areas. The asymmetric transformation showed poorer performance than the groups with symmetric transformation, especially the asymmetric transformation in the Demons algorithm, with *P* = 0.004 (CC) and *P* = 0.00 (DSC). In the asymmetric transformation, only the Optical Flow algorithm showed acceptable performance. For the mapping direction, the forward direction was significantly better than backward mapping, with *P* = 0.048 (CC) and *P* = 0.009 (DSC). For tissue‐tissue interface areas, the symmetric transformation with an Optical Flow algorithm in the forward mapping (Sym‐OF_FW_) showed the best agreement, with mean CC = 0.99 ± 0.01 in Figure 2A and DSC = 0.89 ± 0.03 in Figure 2B.

### The deformation in air‐tissue interface areas

The experimental air‐tissue interface results were consistent between the control and clinical cases. For the air‐tissue interface areas, in Figure 2A and B, the Optical Flow algorithm in both the asymmetric and symmetric transformations showed better performance than the Demons algorithm, with *P* = 0.004 (CC) and *P* = 0.00 (DSC). The Demons algorithm demonstrated poor performance in the asymmetric transformation with both forward and backward mapping.

The DIR accuracy results of the air‐tissue interface areas in cubic no. 7–12 and the clinical cases in Figure 2A and B showed the best performance in asymmetric transformation with Optical Flow algorithms in the backward mapping (Asy‐OF_BW_) a mean CC = 0. 97 ± 0.03 and DSC = 0.79 ± 0.11. The mapping direction was not significantly different; the CC (*P* = 0.707) and DSC (*P* = 0.392) of the forward mapping direction were slightly better than those of the backward mapping.

**Figure 3:**
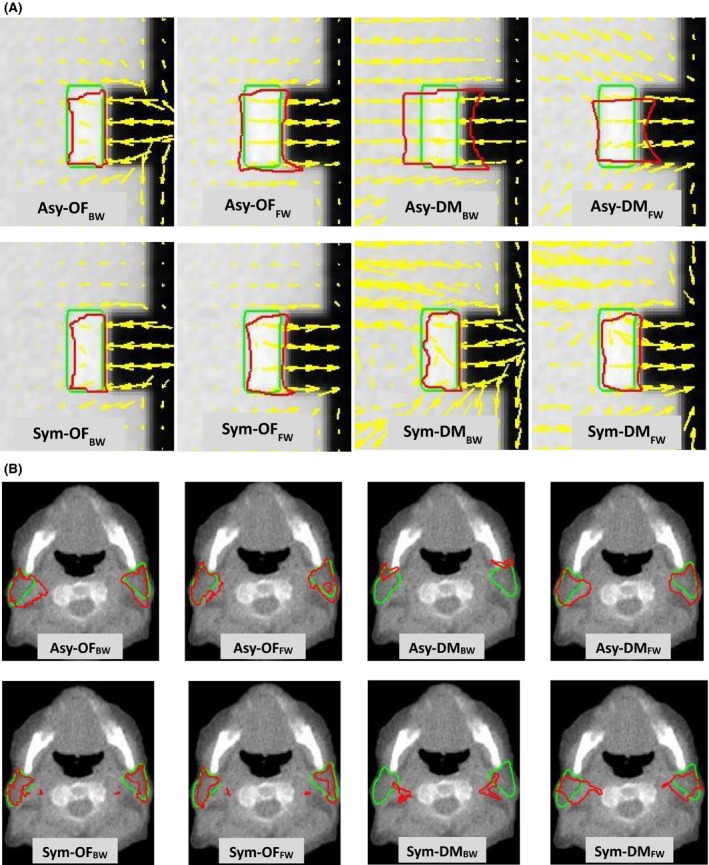
The DVF (yellow arrows) used to identify the motion, the reference contours (green line) and automatic contours using eight DIR methods^†^ (red line) were compared in (A) cubic phantom #8 and (B) the parotid glands of an NPC patient. ^†^Asy‐OF_BW_, asymmetric transformation with the Horn and Schunck optical flow algorithm and backward mapping; Asy‐OF_FW_, asymmetric transformation with the Horn and Schunck optical flow algorithm and forward mapping; Asy‐DM_BW_, asymmetric transformation with the Demon algorithm and backward mapping; Asy‐DM_FW_, asymmetric transformation with the Demon algorithm and forward mapping; Sym‐OF_BW_, symmetric transformation with the Horn and Schunck optical flow algorithm and backward mapping; Sym‐OF_FW_, symmetric transformation with the Horn and Schunck optical flow algorithm and forward mapping; Sym‐DM_BW_, symmetric transformation with the Demon algorithm and backward mapping; Sym‐DM_FW_, symmetric transformation with the Demon algorithm and forward mapping.

**Figure 4:**
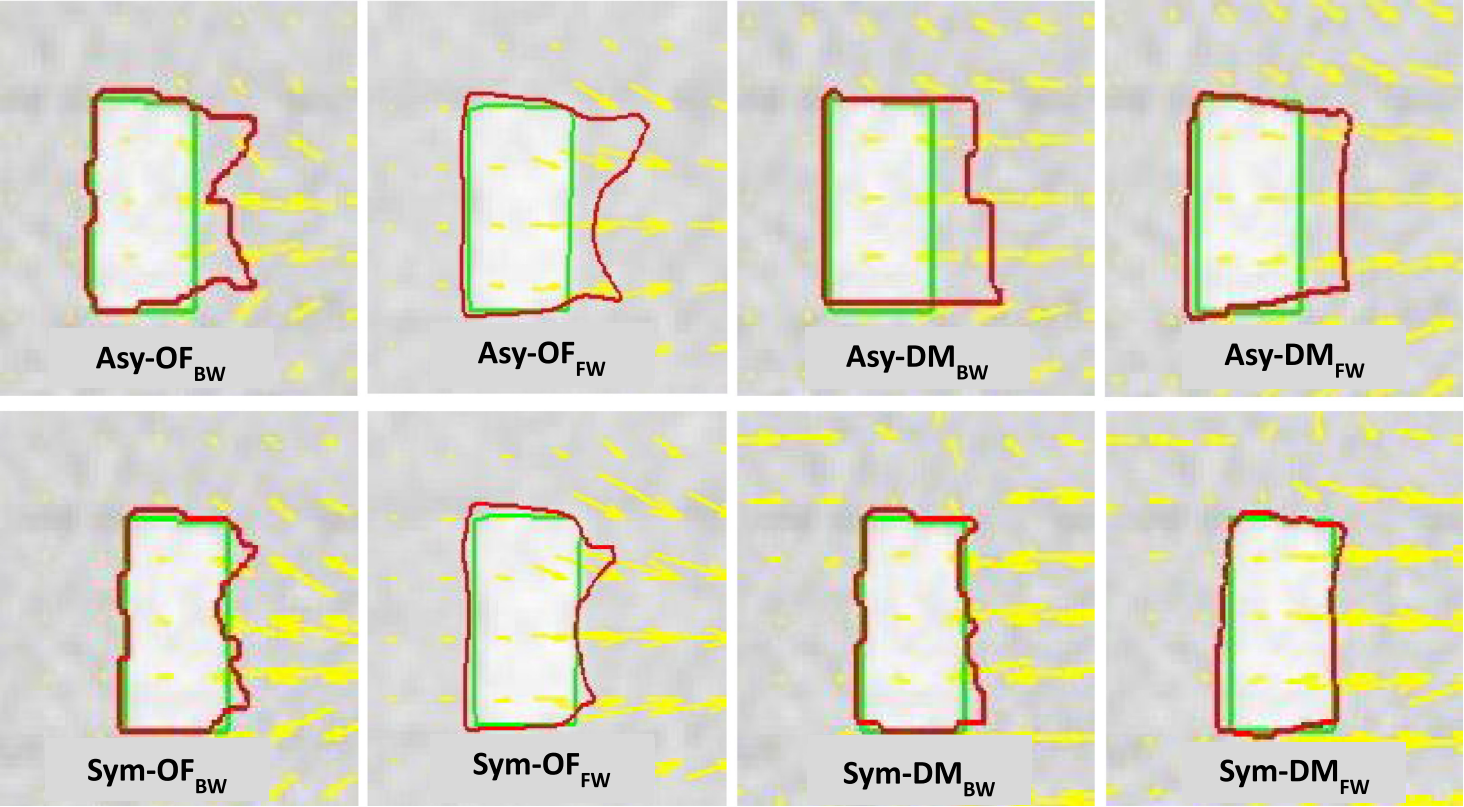
The DVF (yellow arrows) used to identify the motion, the reference contours (green line) and automatic contours using eight DIR methods^†^ (red line) were compared in an acrylic shape insert in cubic phantom #1. ^†^Asy‐OF_BW_, asymmetric transformation with the Horn and Schunck optical flow algorithm and backward mapping; Asy‐OF_FW_, asymmetric transformation with the Horn and Schunck optical flow algorithm and forward mapping; Asy‐DM_BW_, asymmetric transformation with the Demon algorithm and backward mapping; Asy‐DM_FW_, asymmetric transformation with the Demon algorithm and forward mapping; Sym‐OF_BW_, symmetric transformation with the Horn and Schunck optical flow algorithm and backward mapping; Sym‐OF_FW_, symmetric transformation with the Horn and Schunck optical flow algorithm and forward mapping; Sym‐DM_BW_, symmetric transformation with the Demon algorithm and backward mapping; Sym‐DM_FW_, symmetric transformation with the Demon algorithm and forward mapping.

Figure 3 illustrates the deformation in air‐tissue interface areas. The cubic phantom was filled with acrylic material #8 as shown in Figure 3A for rigid deformation; an NPC case is shown in Figure 3B for non‐rigid deformation. Both asymmetric and symmetric transformations using the Optical Flow algorithm demonstrated better performance than those using the Demons algorithm. However, as shown in Figure 4, the symmetry with both Optical Flow and Demons showed better performance in the tissue‐tissue interface areas.

## Discussion

The agreement of the automatically deformed contour from the eight DIR methods used in this study with the reference structure was shown to be dependent on the areas of interest. The accuracy of DIR in tissue‐tissue interface areas showed a higher CC and DSC than the air‐tissue interface areas, with *P* = 0.054 (CC) and *P* = 0.143 (DSC). Regarding asymmetric transformation, only the Optical Flow algorithm showed acceptable performance, as the asymmetric transformation does not estimate the inverse transformation. The results were biased by the choice of the target domain, in contrast to symmetric transformation that uses a method for simultaneously estimating both the forward and backward transformations.^8^

**Figure 5:**
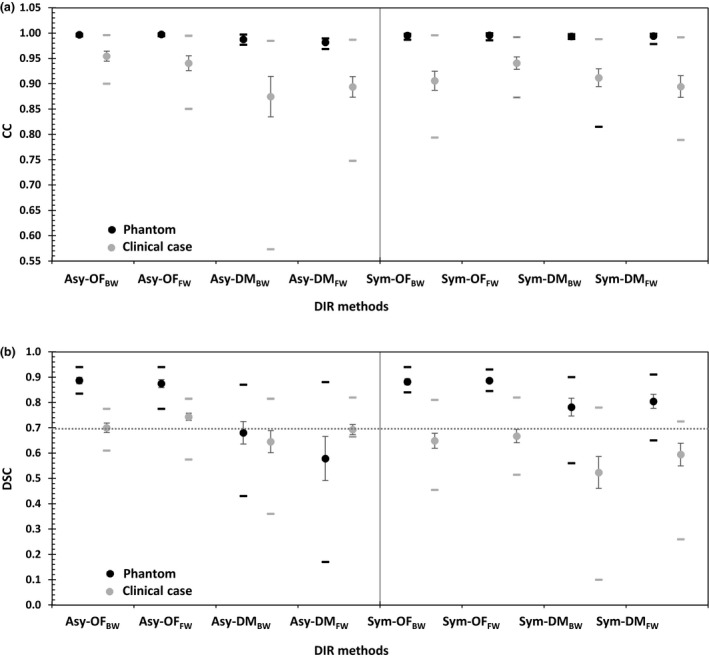
The mean (circles), standard error (vertical lines) and range (horizontal lines) of the (A) correlation coefficient (CC) and (B) Dice's similarity coefficient (DSC) for eight DIR methods^†^ in the phantom and clinical case investigation. ^†^Asy‐OF_BW_, asymmetric transformation with the Horn and Schunck optical flow algorithm and backward mapping; Asy‐OF_FW_, asymmetric transformation with the Horn and Schunck optical flow algorithm and forward mapping; Asy‐DM_BW_, asymmetric transformation with the Demon algorithm and backward mapping; Asy‐DM_FW_, asymmetric transformation with the Demon algorithm and forward mapping; Sym‐OF_BW_, symmetric transformation with the Horn and Schunck optical flow algorithm and backward mapping; Sym‐OF_FW_, symmetric transformation with the Horn and Schunck optical flow algorithm and forward mapping; Sym‐DM_BW_, symmetric transformation with the Demon algorithm and backward mapping; Sym‐DM_FW_, symmetric transformation with the Demon algorithm and forward mapping.

In individual cases, the results were consistent with the phantom and clinical case investigation in air‐tissue interface areas, and the asymmetric transformation with an Optical Flow algorithm and backward mapping (Asy‐OF_BW_) in Figure 5A showed the best agreement, with a mean CC = 0.99 ± 0.01 (phantom) and 0.95 ± 0.03 (clinical case). For the DSC analysis, the asymmetric transformation with an Optical Flow algorithm and backward mapping (Asy‐OF_BW_) also showed the best performance in the phantom, with DSC = 0.89 ± 0.04. The asymmetric transformation with an Optical Flow algorithm and forward mapping (Asy‐OF_FW_) in Figure 5B showed the best agreement with mean DSC = 0.74 ± 0.05 for clinical case.

The accuracy of MVCT image registration is limited by low image contrast. Yeo et al.^6^ assessed the accuracy of 12 DIR algorithms in DIRART software and quantitatively examined low‐contrast regions by developing a deformable gel (DEFGEL). The greatest accuracy was exhibited by the *original Horn and Schunck* optical flow algorithm and the *modified demons* algorithm exhibited the greatest error. Varadhan et al.^13^ described a framework to test the accuracy of DIR using the B‐spline and diffeomorphic demons algorithms with forward and inverse directions. For head and neck study sets, the mean DSC for diffeomorphic demons was 0.74. The diffeomorphic demons algorithm led to gross errors in structures affected in contrast variation. Rigaud et al. compared the performance of ten DIR approaches using different registration methods (Demons or B‐spline free‐form deformation (FFD)), pre‐processing, and similarity metrics. The most effective DIR methods were demons with the mutual information metric and filtered CTs. The mean DSC for Demons with original CTs with the mean square error metric was 0.75 for the parotid gland, showing that the choice of the metric or image pre‐processing was at least as important as the registration method.^15^

This study describes the application of known deformations on any image data set to evaluate the accuracy and limitations of a DIR algorithm used in radiation oncology. The techniques of using phantom and clinical MVCT images allowed for verification of a variety of the deformation methods for DIR quality assurance. The acceptable results for the volume matching using Dice's similarity coefficient should be 0.70 or greater for application in adaptive radiotherapy.^14^ Potential clinical applications include, DIR use for creating cumulative doses by tracking the dose to the tissue voxels throughout a course of treatment to evaluate the dosimetric impact in adaptive radiotherapy.

## Conclusion

The differences in deformation algorithms, transformation frameworks and choice of the target domain for generating the mapping direction affect the accuracy of a DVF. Moreover, the accuracy of DIR depends on the interface areas of deformation. Regarding the air‐tissue interface, both the phantom and clinical cases showed that the asymmetric transformation in Optical Flow algorithms was superior. The symmetric transformation in the Demons algorithm showed an advantageous deformation method in the tissue‐tissue interface area. In the intensity correlation and volume overlapping analysis, the DIR methods showed satisfactory volume matching of greater than 0.70 in the DSC analysis. The methods can yield acceptable results for implementation in adaptive radiotherapy.

## Conflict of Interest

The authors declare no conflict of interest.
